# *Rhodotorula mucilaginosa* Fungemia, a Rare Opportunistic Infection without Central Venous Catheter Implantation, Successfully Treated by Liposomal Amphotericin B

**DOI:** 10.1155/2022/7830126

**Published:** 2022-06-03

**Authors:** Ryuichi Hirano, Tatsuro Mitsuhashi, Katsuyoshi Osanai

**Affiliations:** ^1^Division of Infection Control, Aomori Prefectural Central Hospital, 2-1-1 Higashi-tsukurimichi, Aomori, Japan Postal Code:030-8553; ^2^Division of Pharmacy, Aomori Prefectural Central Hospital, 2-1-1 Higashi-tsukurimichi, Aomori, Japan Postal Code:030-8553

## Abstract

**Background:**

Fungemia due to *Rhodotorula mucilaginosa* is rare and highly resistance to antifungal therapy. Since most cases of *R. mucilaginosa* fungemia are attributed to medical devices, limited information is currently available on infection without central venous catheter (CVC) implantation. We herein report a case of *R. mucilaginosa* fungemia without implantation of CVC, successfully treated by liposomal amphotericin B (L-AMB). *Case Presentation*. An 81-year-old man with a history of chronic obstructive lung disease and rheumatoid arthritis was admitted with dyspnea and fever. The present case had no previous history of CVC implantation. Candidemia was suspected based on yeast and salmon-pink colonies in blood cultures, and thus, micafungin (MCFG) was administered. The isolated yeast was identified as *R. mucilaginosa*, which exhibited resistance to MCFG. Therefore, antifungal therapy was changed to L-AMB. The sterile blood culture and defervescence were observed from the initiation of L-AMB.

**Conclusion:**

Although the obvious entry point was unclear, long-term immunosuppressive therapy for RA may have damaged the gastrointestinal tract, which leading to the bacterial translocation of *R. mucilaginosa*. An early class switch to L-AMB was effective. Physicians need to consider the administration of L-AMB in cases suspected of *R. mucilaginosa* fungemia following the detection of salmon-pink colonies in blood cultures.

## 1. Introduction

*Rhodotorula* species are commensal yeasts with similar bacteriological characteristics to Cryptococcaceas [1]. These species have been isolated from a number of medical devices, including flexible bronchoscopes and central venous catheter (CVC) tips, due to their strong affinity for plastics and high biofilm-forming ability [2–4]. Salmon-pink colonies are a microscopic characteristic of these species [1]. *Rhodotorula* species are less virulent than *Candida* species but have been reported to cause fungemia in patients with immunodeficiency as well as malignancy [5]. Catheter-related bloodstream infections (CRBSI) are some of the most common infections caused by *Rhodotorula* species, in addition to meningitis and endophthalmitis [1, 5]. Although the removal of CVC is beneficial for patients with CRBSI due to *Rhodotorula* species, there have been few reports among patients without implantation of CVC [6]. We herein report a case of *R. mucilaginosa* fungemia without CVC that was successfully treated by liposomal amphotericin B (L-AMB).

## 2. Case Presentation

**Figure 1:**
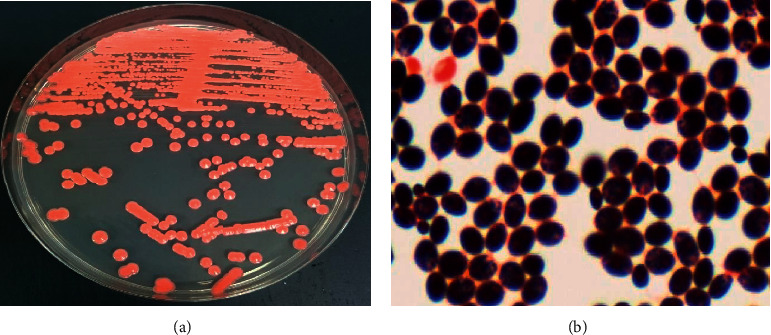
(a) *Rhodotorula mucilaginosa* colonies in Sabrouraud medium. (b) Gram stain of the isolate from blood culture.

An 81-year-old man with a history of chronic obstructive lung disease (COPD) and rheumatoid arthritis (RA) was admitted to our hospital with dyspnea, cough, and fever. The patient was receiving prednisolone 5 mg/day, salazosulfapyridine 1000 mg/day, and clarithromycin 400 mg/day for COPD and RA. His vital signs were as follows: blood pressure 142/119 mmHg, body temperature 37.7°C, respiratory rate 20 breaths/min, and arterial oxygen saturation 88% (4 L). Chest X-ray showed decreased permeability in the lungs, and an infiltrative shadow was detected on chest computed tomography. Laboratory findings at admission were as follows: white blood cell count 9,100/mm^3^, C-reactive protein 12.7 mg/dL, procalcitonin 0.14 ng/mL, serum creatinine 0.62 mg/dL, serum potassium 3.7 mEq/L, and serum (1⟶3)-*β*-D glucan 18.1 pg/mL. The patient was diagnosed with the acute exacerbation of COPD based on pneumonia, and meropenem (MEPM) and methylprednisolone were administered from day 1. *Pseudomonas aeruginosa* and *Haemophilus influenzae* were isolated from a sputum culture submitted at admission. Candidemia was suspected based on yeast and salmon-pink colonies in a blood culture submitted at admission (Figure 1), and thus, micafungin (MCFG) 150 mg QD was administered from day 6. No findings suggestive of endophthalmitis were observed based on an ophthalmological examination. On day 8, the yeast isolated from the blood culture was identified as *Rhodotorula* species according to the VITEK 2 YST ID Card (SYSMEX; bioMerieux, Lyon, France). An antifungal susceptibility test was performed using the yeast-like fungal drug susceptibility kit ASTY (Kyokuto Pharmaceutical Industrial, Tokyo, Japan). The minimum inhibitory concentrations of each antifungal agent are shown in Table 1. According to the guidelines, *Rhodotorula* species are considered to be intrinsically resistant to azoles and echinocandins [6]. The isolated yeast was identified as *R. mucilaginosa* by a sequencing analysis of ITS and the D1/D2 region of ribosomal DNA using panfungal primers [7, 8]. Based on these findings, the patient was diagnosed with fungemia due to *R. mucilaginosa*, and antifungal therapy was changed from MCFG to L-AMB 3 mg/kg QD on day 8. In addition to defervescence, a sterile blood culture was noted on day 10. Due to a decrease in the serum level of potassium to 2.6 (mEq/L), potassium supplementation was initiated on day 17. The serum level of potassium increased to 4.3 (mEq/L) by day 23. L-AMB therapy was continued for 17 days and no increase in the serum level of creatinine was observed. The patient was discharged on day 64 with no signs of relapse of the infection.

## 3. Discussion

We herein described a case of fungemia due to *R. mucilaginosa* that was successfully treated with L-AMB. Although *R. mucilaginosa* fungemia is a rare opportunistic infection, it is the fourth most common causative species of fungemia, except for *Candida* species [9]. In addition to their high resistance to antifungal agents, *Rhodotorula* species cause approximately 50% of cases of non-*Candida* and *Cryptococcus* fungemia in patients with malignancy [10]. Therefore, further information is needed to manage this fungal infection, particularly in immunocompromised hosts.

A previous study identified *R. mucilaginosa* as the most common species causing fungemia due to *Rhodotorula* species [11]. The overall mortality rate was approximately 12% [11]. Malignancy, autoimmune disease, immunosuppressive therapy, and corticosteroid use have been identified as risk factors for the onset of fungemia due to *Rhodotorula* species [1, 5]. The present case had received prednisolone and salazosulfapyridine to treat RA, which may have been the causative factor for the onset of *R. mucilaginosa* fungemia. Although the implantation of CVC has been reported as the most common risk factor for fungemia due to *R. mucilaginosa*, the present case had no previous history of CVC implantation [1, 5, 12]. Besides CVC, bacterial translocation associated with an injured gastrointestinal tract is an entry point for *R. mucilaginosa*, a gastrointestinal bacterium that resides in the colon [1]. Although the obvious entry point was unclear in the present case, long-term immunosuppressive therapy for RA may have damaged the gastrointestinal tract, leading to the bacterial translocation of *R. mucilaginosa.*

In the present case, serum (1⟶3)-*β*-D glucan was 18.1 pg/mL, which was not significantly elevated despite fungemia. The results obtained in the present case were consistent with previous findings showing no increase in serum (1⟶3)-*β*-D glucan at the onset of fungemia due to *R. mucilaginosa* [13]. The fungal characteristics of *Rhodotorula* species are similar to those of *Cryptococcus* species. Among patients with *Cryptococcus* fungemia, (1⟶3)-*β*-D glucan did not increase because the cell wall of *Cryptococcus* species comprises (1⟶6)-*β*-D glucan [13]. The composition of the cell wall of *R. mucilaginosa* may contribute to the lack of an increase in serum (1⟶3)-*β*-D glucan in patients with *R. mucilaginosa* fungemia; however, the underlying mechanisms have not yet been elucidated in detail. Further studies on serum (1⟶3)-*β*-D glucan levels in *R. mucilaginosa* fungemia are warranted because limited information is currently available on this biomarker at the onset of disease. The present case supports the importance of blood culture tests for patients suspected of invasive fungal infection.

*R. mucilaginosa* isolated from the present case was resistant to echinocandins, e.g., MCFG and CPFG, whereas it seemed to be susceptible to AMB. Susceptibility patterns in the present case were consistent with the findings of previous antifungal susceptibility surveillance for *Rhodotorula* species [14]. An early class switch to L-AMB contributed to bacterial clearance and defervescence in the present case because *Rhodotorula* species are intrinsically resistant to azoles and echinocandins [6].

Fungemia due to *R. mucilaginosa* has been defined as a rare fungal infection according to the guidelines of the European Society of Clinical Microbiology and Infectious Disease (ESCMID) [15]. According to the latest guidelines, AMB preparations are the preferred treatment option based on susceptibility patterns, and source control, e.g., the removal of CVC is strongly recommended in patients with a CVC implanted [6]. In the present case, there were no sites of infection that needed removal because a CVC was not implanted. Therefore, the administration of L-AMB was essential for treatment.

Regarding the present case, laboratories with limited experience in mycology may not be aware that pink colonies are not a characteristic of *Candida* species, which results in a delay in the initiation of L-AMB therapy. *Rhodotorula* and *Sporobolomyces* species both form characteristic pink colonies, and the guidelines recommended the avoidance of echinocandin therapy for patients with suspected infections by these yeasts [6]. Fortunately, the delay in initiating L-AMB therapy did not adversely affect the outcome of the present case. This may be attributed to the lower virulence of *R. mucilaginosa* than that of more common fungal pathogens, e.g., *Candida* species [5]. The present case supports the importance of alerting physicians of expected susceptibility patterns in patients with laboratory results showing the characteristic colony findings of yeasts.

Hypokalemia and renal dysfunction have been reported as severe side effects of L-AMB [16]. Potassium supplementation is essential for the prevention of hypokalemia because of its high frequency during the administration of L-AMB [17]. In the present case, close monitoring and potassium supplementation effectively prevented the development of hypokalemia during L-AMB therapy. There is currently no established duration for antifungal therapy in patients with *R. mucilaginosa* fungemia. The present case received L-AMB therapy for 14 days after the confirmation of a sterile blood culture test, in accordance with a previous study [18].

## 4. Conclusion

We herein report a case of fungemia due to *R. mucilaginosa* that was successfully treated by L-AMB. Although the obvious entry point was unclear, immunosuppressive therapy with RA potentially damaged the gastrointestinal tract, which may have resulted in the bacterial translocation of *R. mucilaginosa.* Physicians need to consider the administration of L-AMB in immunocompromised cases suspected of *R. mucilaginosa* fungemia following the detection of salmon-pink colonies in blood cultures.

## Figures and Tables

**Table 1:** Minimal inhibitory concentration and susceptibility pattern for *Rhodotorula mucilaginosa* isolated from a blood culture.

Antifungal agents	Minimum inhibitory concentration (*μ*g/mL)
Amphotericin B	0.25
Fluconazole	64
Itraconazole	4
Micafungin	16
Voriconazole	2
Caspofungin	8

## Data Availability

No data were used to support this study.
